# Socioeconomic Inequities in Preemptive Kidney Transplantation and Graft Survival: An Innovative Approach to Identifying Disparities in Kidney Transplantation

**DOI:** 10.1097/TXD.0000000000001734

**Published:** 2024-12-18

**Authors:** Sarah Kizilbash, Chung-II Wi, Madison Roy, Euijung Ryu, Arthur Matas, Vesna Garovic, Samy Riad, Carrie Schinstock, Young Juhn

**Affiliations:** 1 Department of Pediatrics, University of Minnesota, Minneapolis, MN.; 2 Precision Population Science Lab, Department of Pediatric and Adolescent Medicine Mayo Clinic, Rochester, MN.; 3 Precision Population Science Lab, Department of Quantitative Health Science, Mayo Clinic, Rochester, MN.; 4 Department of Surgery, University of Minnesota, Minneapolis, MN.; 5 Division of Nephrology and Hypertension, Department of Internal Medicine, Mayo Clinic, Rochester, MN.; 6 Precision Population Science Lab, Department of Pediatric and Adolescent Medicine/Internal Medicine, Mayo Clinic Rochester and Mayo Clinic Health System, MN.

## Abstract

**Background.:**

The limitations of conventional measures of socioeconomic status (SES) limit our ability to elucidate the role of SES as a key element of social determinants of health in kidney transplantation. This study’s objective was to use an innovative SES measure, the HOUsing-based SES measure (HOUSES) index, to examine the effects of social determinants of health on access to and outcomes of kidney transplantation.

**Methods.:**

Our study included residents of Minnesota (age older than 18 y) who underwent kidney transplantation at a single center between 2010 and 2020. SES was determined using the HOUSES index, categorized into quartiles (Q1 for lower, Q2–Q4 for higher SES). We used mixed-effects multivariable logistic and Cox models to examine the effects of HOUSES on preemptive transplants, pretransplant dialysis duration, and death-censored graft loss, adjusting for covariates.

**Results.:**

Among 1975 eligible patients, 29.4% received preemptive transplants, 34.9% underwent pretransplant dialysis for >3 y, and 15.1% experienced death-censored graft loss for a median follow-up of 7.15 (interquartile range, 4.25–11.38) y. Lower SES recipients (Q1) demonstrated decreased preemptive transplant likelihood (adjusted odds ratio [aOR]: 0.74; 95% confidence interval [CI], 0.57-0.97; *P* = 0.03), longer dialysis duration (>3 y; aOR: 1.43; 95% CI, 1.01-2.03; *P* = 0.046), and higher death-censored graft loss (adjusted hazard ratio 1.36; 95% CI, 1.02-1.12; *P* = 0.036) versus higher SES recipients (Q2–Q4).

**Conclusions.:**

We observed significant socioeconomic disparities in kidney transplant access, dialysis duration, and graft survival. The HOUSES index may be a promising tool for individual-based targeted interventions as it identifies SES on an individual rather than an area-level basis.

## Graphical abstract

**Figure:**
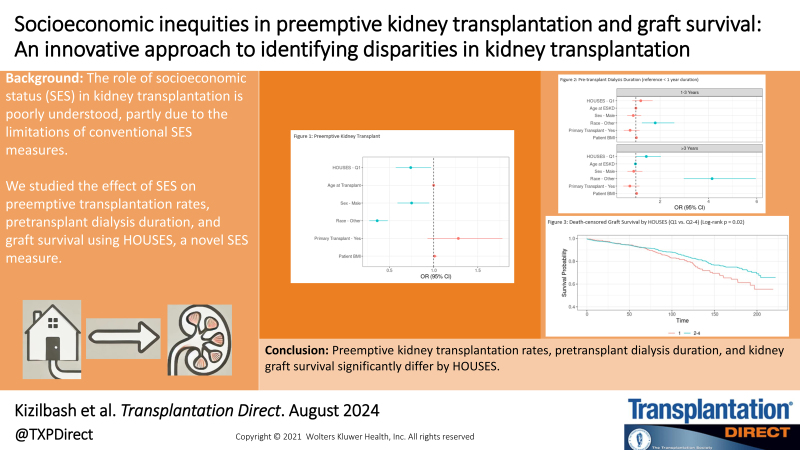

Kidney transplantation is associated with superior survival and a better quality of life compared with dialysis.^1^ The Kidney Disease: Improving Global Outcomes guidelines recommend preemptive kidney transplants (transplant before dialysis initiation) as the preferred treatment for children and adults with kidney failure. Preemptive transplants offer numerous benefits, including avoidance of surgical placement of dialysis access, dialysis-associated morbidity, improved quality of life, better employment rates, and cost savings for the healthcare system.^2,3^ Furthermore, preemptive transplants consistently demonstrate superior patient and graft survival outcomes compared with transplants performed after a dialysis period.^4-6^ However, the rates of preemptive transplantation in the United States are disproportionately low. In 2020, 130 522 individuals developed kidney failure in the United States, and of these, 83.9% initiated hemodialysis, 12.7% commenced peritoneal dialysis, and only 3.1% underwent a preemptive transplant.^7^ Concerted efforts are needed to improve preemptive transplantation rates, but designing effective interventions requires a deeper understanding of modifiable factors that hinder preemptive transplants.

In addition to ensuring equitable and timely access to kidney transplants, mitigating risk factors for untimely graft loss is paramount for optimizing outcomes. In the last 2 decades, there has been a marked improvement in 1-y graft survival, but long-term graft survival remains suboptimal, with the median graft survival for deceased- and living-donor recipients being 11.7 and 19.2 y, respectively.^8^ By necessitating dialysis or retransplantation, graft loss results in significant morbidity and mortality. Furthermore, it imposes a financial strain on the healthcare system. Therefore, modifiable risk factors that may lend themselves to focused interventions for risk mitigation must be identified. Social determinants of health (SDOH) may be such modifiable factors.^9-13^

Socioeconomic status (SES) is a key element of SDOH. It is defined as one’s capacity to access and use desired resources and can be positively influenced through targeted support and interventions.^14,15^ Studies indicate lower patient and graft survival among kidney transplant recipients with a lower SES (residing in deprived neighborhoods,^16-18^ lower educational attainment^19,20^); however, most studies are restricted because of substantial limitations of the conventional SES measures. Studies using national registries frequently use area-level SES measures, which may misclassify individual-level SES in up to 43% of instances.^21,22^ Using area-level measures of SES as proxies for individual-level SES may lead to inaccurate estimation of the association between SES and health outcomes.^22-25^ Conversely, accurate income-based, individual-level SES measures are challenging to obtain as participants often decline to respond or report inaccurate income.^26^ Considering the limitations of existing measures, improved SES measures are needed to investigate the role of SES in kidney transplantation.

To overcome the limitations of prior studies, we used the individual HOUsing-based Socioeconomic Status (termed HOUSES hereafter) index to examine the effects of SES on transplant access and outcomes.^27^ HOUSES is generated by linking address information from electronic health records or other data sources to publicly available real property data. It captures the household’s net wealth and income, social and environmental resources, and the effects of the building features on health.^27^ Our prior study, based on a smaller sample size, demonstrated an association between HOUSES and the risk of graft loss in patients residing in Olmsted County, Minnesota.^25^ The present study aimed to validate the association between HOUSES and the risk of graft loss within a larger and more diverse transplant population in a metropolitan city. Furthermore, the study aimed to investigate the association of HOUSES with access to preemptive transplantation and pretransplant dialysis duration among a cohort of transplant recipients, which was not evaluated in our previous study. The disparities in preemptive transplantation and pretransplant dialysis duration have been described for racial minorities and Medicare beneficiaries.^28,29^ We hypothesized fewer preemptive transplants, longer pretransplant dialysis duration, and increased hazards of graft loss in transplant recipients with lower SES as measured by HOUSES. If these hypotheses are supported, the study will offer an innovative approach for identifying individuals with a reduced likelihood of preemptive transplants and an increased risk of graft loss, paving a path for individual-level, targeted interventions to optimize access and outcomes.

## MATERIALS AND METHODS

### Study Design

This retrospective cohort study evaluated the differential effect of individual-level SES, as measured by HOUSES, on access to preemptive transplants, pretransplant dialysis duration, and the risk of death-censored graft loss. The Institutional Review Board at the University of Minnesota and the Mayo Clinic approved this study, which was performed in adherence to the Declaration of Helsinki. The clinical and research activities being reported are consistent with the Principles of the Declaration of Istanbul as outlined in the “Declaration of Istanbul on Organ Trafficking and Transplant Tourism.”

### Study Population and Cohort

Using a prospectively maintained, Institutional Review Board–approved, solid organ transplant database at the University of Minnesota, we identified all adult kidney transplant recipients transplanted between January 1, 2010, and January 1, 2020, who were residing in Minnesota at the time of the transplant. The transplant database contains posttransplant, peritransplant, and minimal pretransplant data on recipients and donors. Information on demographics, pretransplant comorbidities, transplant surgery variables, posttransplant complications, and outcomes are captured via manual chart abstraction and electronic data transfer from the electronic medical record.

### HOUSES and Psychometric Properties

HOUSES is a robust, individual-level SES measure computed from 4 variables (number of bedrooms, number of bathrooms, square footage of the unit, and estimated building value) ascertained from the county Assessor’s office based on home addresses, which is normalized within each county.^27^ Each property item corresponding to an individual’s address is standardized into a z-score and aggregated into an overall z-score for the 4 items such that a higher HOUSES score indicates higher SES. Ownership was one of the 14 variables included in the original study for HOUSES development,^27^ but not selected within the same factor with the other 4 variables of the final HOUSES index. The results of adding ownership were not significantly different from the parsimonious model based on 4 variables. HOUSES was then divided into quartiles (Q1 indicating the lowest SES). Construct validity of HOUSES has been extensively assessed using health outcomes known to be associated with SES, as shown in **Appendix Tables 1 and 2 (SDC,** http://links.lww.com/TXD/A718). To make HOUSES scalable, an automated cloud-based platform was recently established, making the HOUSES formulation process automatic and making HOUSES available to nationwide users. HOUSES has been extensively validated and reported in 41 publications (**Appendix Tables 1 and 2, SDC,** http://links.lww.com/TXD/A718), showing its associations with 62 outcomes in about 1.5 million patients. Thus, HOUSES is a validated, standardized, objective, patient contact-free, and nationwide individual SES measure.

For this study, we retrieved recipient addresses from the University of Minnesota transplant registry at the time of transplantation to compute HOUSES for each recipient by following the HOUSES index computing algorithm. Hence, HOUSES provided “the individual-level SES at the time of transplant for all recipients in the study.”

### Other Study Variables

In addition to the SES variable described above, we retrieved the following data from the registry: age at the time of transplant, sex, race, pretransplant dialysis (transplant after a period of dialysis or a preemptive transplant), donor source (deceased or living), prior transplant, HLA mismatch, recipient’s body mass index (BMI), posttransplant dialysis, date and cause of graft loss (defined as a return to dialysis or retransplantation), and date of death.

We also retrieved data on education level (some college education versus high school or less), insurance type (private, Medicaid, Medicare and Choice, Medicare – fee for service, and other), and work income (yes or no) at the time of transplant.

### Study Outcomes

Our outcomes of interest included preemptive deceased- and living-donor kidney transplants and death-censored graft loss. We also examined pretransplant dialysis duration. Preemptive transplants were defined as transplants before the need to initiate dialysis. For death-censored graft loss, patients were followed from the transplant date to the earliest of the dates of graft loss, death, or the end of study follow-up. For a secondary outcome, pretransplant dialysis duration was defined as the interval between the first day of chronic dialysis and kidney transplantation. We classified pretransplant dialysis duration into 3 categories: <1 y, 1–3 y, and >3 y, based on previous research findings showing increased mortality with dialysis duration of 1–3 y and >3 y compared with the duration of <1 y.^30^

### Statistical Analysis

Descriptive statistics were used to summarize the characteristics of the cohort by dichotomous HOUSES quartiles (Q1 versus Q2–Q4). This approach is consistent with our earlier study, facilitating the identification of socially and medically underserved transplant recipients.^25^ We first assessed the univariate association of HOUSES with patient characteristics at the time of the transplant using ANOVA for continuous variables and the Pearson chi-square test for categorical variables. To be consistent with our prior analytic approach, we used the Kaplan-Meier method and the log-rank test to compare graft survival between HOUSES Q1 and Q2–Q4.^25^ We also examined the effect of HOUSES on preemptive transplants, pretransplant dialysis duration, and death-censored graft survival using HOUSES quartiles. We evaluated the association between HOUSES and death-censored graft loss using a mixed-effects Cox regression model having counties as random terms (for modeling HOUSES, which is a relative SES within a given county) and age at transplant (as groups), sex, race, pretransplant dialysis, donor type, HLA mismatch, and primary transplant as fixed terms. We used mixed-effects multivariable logistic models (county as a random effect) to evaluate the effect of HOUSES on (1) preemptive transplantation adjusting for age at kidney loss, sex, race, prior transplantation, and BMI, and (2) pretransplant dialysis duration (categorical variable with 3 levels: <1 y, 1–3 y, >3 y). Covariates were selected for multivariable models based on their previously known association with the outcomes of interest.^31^ All analyses were performed using R (R Core Team (2021).^32^

## RESULTS

### Sociodemographic and Clinical Characteristics

Between January 1, 2010, and January 1, 2020, 2090 adult recipients underwent kidney transplantation at the University of Minnesota and lived in Minnesota at the time of transplant. Of these, 110 were excluded from the analysis due to missing house addresses, and 5 were excluded for unknown dialysis history. The remaining 1975 recipients were followed for a median of 7.15 (25th and 75th percentiles: 4.25–11.38) y. The baseline and transplant characteristics of the study population, stratified by HOUSES, are shown in Table 1. Overall, the mean age at transplant was 51.7 y (range, 21.0–81.8). Of the recipients, 60.6% were men, and 76.6% were White.

**TABLE 1.** Sociodemographic and transplant characteristics by HOUSES

Variables	Low SES (HOUSES Q1) (N = 588)	Higher SES (HOUSES Q2–Q4) (N = 1387)	*P*
Age at kidney failure			0.024
Mean (SD)	47.39 (14.23)	49.38 (14.25)	
Age at transplant			0.01^*a*^
Mean (SD)	50.60 (13.35)	52.22 (13.45)	
Sex			0.77^*b*^
Female	229 (38.9%)	550 (39.7%)	
Race			<0.001^*b*^
Non-Hispanic White	399 (67.9%)	1114 (80.3%)	
Black	110 (18.7%)	96 (6.9%)	
Asian	47 (8.0%)	144 (10.4%)	
American Indian or Alaska Native	19 (3.2%)	27 (1.9%)	
Other	13 (2.2%)	6 (0.4%)	
Education level			0.006^*b*^
At least some college	253 (43.0%)	672 (48.4%)	
High school or less	243 (41.3%)	464 (33.5%)	
None	12 (2.0%)	22 (1.6%)	
Unknown	80 (13.6%)	229 (16.5%)	
Insurance			<0.001^*b*^
Private	212 (36.1%)	682 (49.2%)	
Medicaid	36 (6.1%)	62 (4.5%)	
Medicare and choice	75 (12.8%)	150 (10.8%)	
Medicare fee for service	252 (42.9%)	479 (34.5%)	
Other	13 (2.2%)	14 (1.0%)	
Work income at the time of transplant			0.088^*b*^
No	359 (61.1%)	806 (58.1%)	
Unknown	10 (1.7%)	47 (3.4%)	
Yes	219 (37.2%)	534 (38.5%)	
Time on waitlist, y			<0.001^*a*^
Missing, n	37	138	
Mean (SD)	2.39 (2.29)	2.03 (1.96)	
Time on dialysis			0.002^*a*^
Missing, n	200	583	
<1 y	89 (22.9%)	245 (30.5%)	
1–3 y	139 (35.8%)	303 (37.7%)	
>3 y	160 (41.2%)	256 (31.8%)	
Donor type			<0.001^*b*^
Deceased	367 (62.4%)	726 (52.3%)	
Primary transplant			0.78^*b*^
No	104 (17.7%)	238 (17.2%)	
HLA mismatch			0.88^*a*^
Missing, n	23	46	
Mean (SD)	3.52 (1.85)	3.53 (1.77)	
Recipient’s pretransplant BMI			0.17^*a*^
Missing, n	128	352	
Mean (SD)	27.27 (5.26)	26.89 (4.81)	
Cyclosporine			0.492^*b*^
Missing, n	2	1	
No	420 (71.7%)	972 (70.1%)	
Yes	166 (28.3%)	414 (29.9%)	
Tacrolimus			0.379^*b*^
Missing, n	2	1	
No	183 (31.2%)	461 (33.3%)	
Yes	403 (68.8%)	925 (66.7%)	
Mycophenolate			0.069^*b*^
Missing, n	2	1	
No	48 (8.2%)	151 (10.9%)	
Yes	538 (91.8%)	1235 (89.1%)	
Azathioprine			0.335^*b*^
Missing, n	2	1	
No	583 (99.5%)	1373 (99.1%)	
Yes	3 (0.5%)	13 (0.9%)	
Thymoglobulin induction			0.412^*b*^
Missing, n	0	2	
No	20 (3.4%)	58 (4.2%)	
Yes	568 (96.6%)	1327 (95.8%)	
Alemtuzumab induction			0.657^*b*^
Missing, n	0	2	
No	581 (98.8%)	1365 (98.6%)	
Yes	7 (1.2%)	20 (1.4%)	
Steroid-inclusive maintenance immunosuppression			0.870^*b*^
No	461 (78.4%)	1092 (78.7%)	
Yes	127 (21.6%)	295 (21.3%)	
eGFR at the time of transplant			
Mean (SD)	16.71 (7.67)	16.21 (7.90)	0.513^*a*^
Cause of graft loss			0.16^*b*^
Acute rejection	14 (14.0%)	17 (8.5%)	
Chronic rejection	34 (34.0%)	46 (23.1%)	
Graft thrombosis	0 (0.0%)	4 (2.0%)	
Infection	5 (5.0%)	14 (7.0%)	
Primary graft loss	13 (13.0%)	30 (15.1%)	
Recurrent disease	4 (4.0%)	10 (5.0%)	
Other	30 (30.0%)	78 (39.2%)	

Values are presented in mean (SD) or n (%).

^*a*^Linear model ANOVA.

^*b*^Pearson’s chi-square test.

BMI, body mass index; eGFR, estimated glomerular filtration rate; HOUSES, HOUsing-based SocioEconomic Status measure.

In an unadjusted analysis, lower SES, as measured by lower HOUSES quartiles (Q1), was associated with a younger age at transplant (Q1 versus Q2–Q4: 50.6 versus 52.2 y; *P* = 0.014), a higher likelihood of being Black (Q1 versus Q2–Q4: 18.7% versus 6.9%; *P* < 0.001), a longer waitlist time (Q1 versus Q2–Q4: 2.4 versus 2.0; *P* < 0.001), more likely to have pretransplant dialysis duration of >3 y (Q1 versus Q2–Q4: 41.2% versus 31.8%; *P* = 0.002), and a lower likelihood of a living-donor transplant (Q1 versus Q2–Q4: 37.6% versus 47.7%; *P* < 0.001. Low SES, defined as HOUSES Q1, was not associated with HLA mismatch, pretransplant BMI, or history of a prior transplant. Furthermore, kidney transplant recipients belonging to Q1 were less likely to have a college education (Q1 versus Q2–Q4: 43.0% versus 48.4%; *P* = 0.006) and less likely to have private insurance (Q1 versus Q2–Q4: 36.1% versus 49.2%; *P* < 0.001). We found no significant differences in the causes of graft loss, including rejection, infection, graft thrombosis, primary graft loss, or recurrent disease, between Q1 and Q2–Q4 recipients (*P* = 0.16; Table 1).

### Access to Preemptive Transplantation

As shown in Table 2, Q1 recipients were less likely to receive a preemptive transplant compared with Q2–Q4 recipients (odds ratio [OR]: 0.66; 95% confidence interval [CI], 0.53-0.83) on unadjusted analysis. Similarly, Q1 (OR: 0.51; 95% CI, 0.38-0.67), Q2 recipients (OR: 0.63; 95% CI, 0.48-0.84), and Q3 recipients (OR: 0.72; 95% CI, 0.55-0.96) were less likely to receive a preemptive transplant compared with Q4 recipients.

**TABLE 2.** Sociodemographic characteristics and their association with preemptive transplantation and dialysis duration

	Preemptive transplant OR (95% CI)	Dialysis 1–3 y OR (95% CI)^*a*^	Dialysis >3 y OR (95% CI)^*a*^
Age at ESKD	1.01 (1.00-1.01)	0.99 (0.98-1.00)	0.97 (0.96-0.98)
Age at transplant	1.01 (1.00-1.01)	1.00 (0.99-1.01)	1.00 (0.99-1.01)
Sex			
Male	**0.80 (0.66-0.97**)	0.99 (0.74-1.34)	0.95 (0.70-1.28)
Race/ethnicity			
Other	**0.37 (0.28-0.47**)	**1.96 (1.40-2.75**)	**4.60 (3.29-6.44**
Education			
High school or less	**0.51 (0.41-0.64**)	**1.63 (1.19-2.24**)	**2.20 (1.60-3.03**)
None	**0.17 (0.04-0.49**)	1.92 (0.47-7.79)	**8.33 (2.44-28.49**)
Unknown	**0.73 (0.55-0.96**)	1.53 (0.98-2.39)	1.37 (0.85-2.20)
Insurance			
Medicaid	**0.43 (0.26-0.68**)	1.04 (0.51-2.11)	**7.83 (4.00-15.31**)
Medicare and choice	**0.18 (0.11-0.26**)	**2.72 (1.62-4.54**)	**19.28 (11.13-33.40**)
Medicare fee for service	**0.21 (0.16-0.26**)	**2.47 (1.79-3.41**)	**11.17 (7.41-16.86**)
Other	**0.42 (0.16-0.95**)	1.37 (0.45-4.15)	**4.55 (1.40-14.81**)
Recipient BMI	1.01 (0.99-1.03)	1.01 (0.98-1.04)	1.01 (0.98-1.04)
HOUSES quartile			
Binary – Q1	**0.66 (0.53-0.83**)	1.26 (0.92-1.72)	**1.70 (1.24-2.32**)
Quartile 3	**0.72 (0.55-0.96**)	1.43 (0.69-2.20)	1.41 (0.89-2.22)
Quartile 2	**0.63 (0.48-0.84**)	1.46 (0.95-2.24)	**1.77 (1.13-2.78**)
Quartile 1	**0.51 (0.38-0.67**)	**1.64 (1.08-2.50**)	**2.38 (1.54-2.38**)

Bolded values indicate statistical significance at *P* < 0.05.

Reference levels: sex – female; race/ethnicity – non-Hispanic White; education – at least some college; insurance – private insurance; HOUSES binary Q2–Q4; HOUSES quartile Q4.

^*a*^The last 2 columns are results from 1 multinomial model with 3 outcomes for time on dialysis: <1 y (reference), 1–3 y (middle column), and >3 y (right column).

BMI, body mass index; CI, confidence interval; ESKD, end-stage kidney disease; HOUSES, HOUsing-based SocioEconomic Status measure; OR, odds ratio.

**FIGURE 1.**
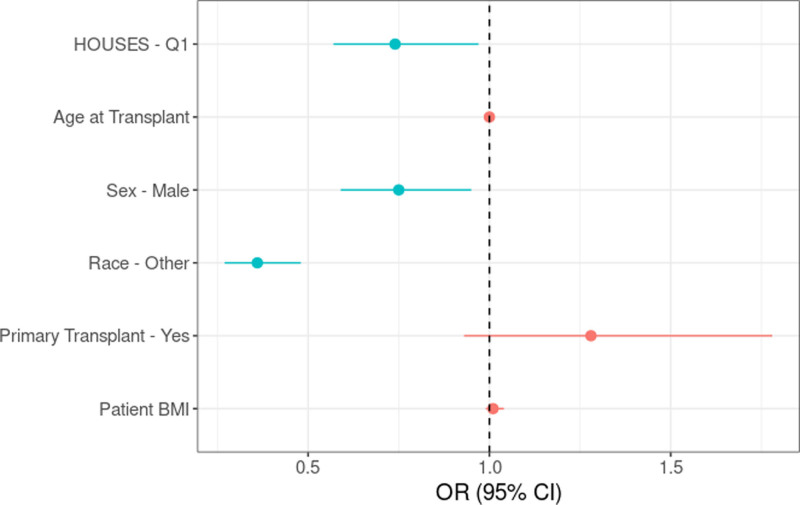
Preemptive kidney transplant. BMI, body mass index; CI, confidence interval; HOUSES, HOUsing-based SocioEconomic Status measure; OR, odds ratio.

After adjusting for age at kidney failure, sex, race, prior transplantation, and recipient BMI, Q1 recipients were less likely to receive a preemptive transplant compared with Q2–Q4 recipients (adjusted OR [aOR]: 1.35; 95% CI, 1.03-1.77; *P* = 0.03; Table 3; Figure 1). When the analysis was performed using the HOUSES quartiles, Q1 (aOR: 0.53; 95% CI, 0.38-0.74; *P* < 0.001), Q2 (aOR: 0.57; 95% CI, 0.40-0.81; *P* = 0.002), and Q3 recipients (aOR: 0.66; 95% CI, 0.47-0.93; *P* = 0.017) had significantly lower odds of receiving a preemptive transplant compared with Q4 recipients (**Table S1, SDC,** http://links.lww.com/TXD/A718).

**TABLE 3.** Preemptive kidney transplantation – adjusted analysis

Variables	OR	95% CI	*P*
HOUSES			
Q1	0.74	0.57-0.97	0.03
Q2–Q4		Reference	
Age at transplant	1.00	0.99-1.01	0.34
Sex			
Female		Reference	
Male	0.75	0.59-0.95	0.02
Race			
Non-Hispanic White		Ref	
Other	0.36	0.27-0.48	<0.001
Primary transplant			
No		Reference	
Yes	1.28	0.93-1.78	0.13
Patient BMI, kg/m^2^	1.01	0.99-1.04	0.37

BMI, body mass index; CI, confidence interval; HOUSES, HOUsing-based SocioEconomic Status measure; OR, odds ratio.

### Pretransplant Dialysis Duration

As shown in Table 2, Q1 recipients were more likely to have been on pretransplant dialysis for >3 y compared with Q2–Q4 recipients (OR: 1.70; 95% CI, 1.24-2.32) on unadjusted analysis. Similarly, Q1 (OR: 2.38; 95% CI, 1.54-2.38) and Q2 recipients (OR: 1.77; 95% CI, 1.13-2.78) were more likely to have been on pretransplant dialysis for >3 y compared with Q4 recipients, but there was no statistically significant difference between Q3 versus Q4 recipients (OR: 1.41; 95% CI, 0.89-2.22).

**FIGURE 2.**
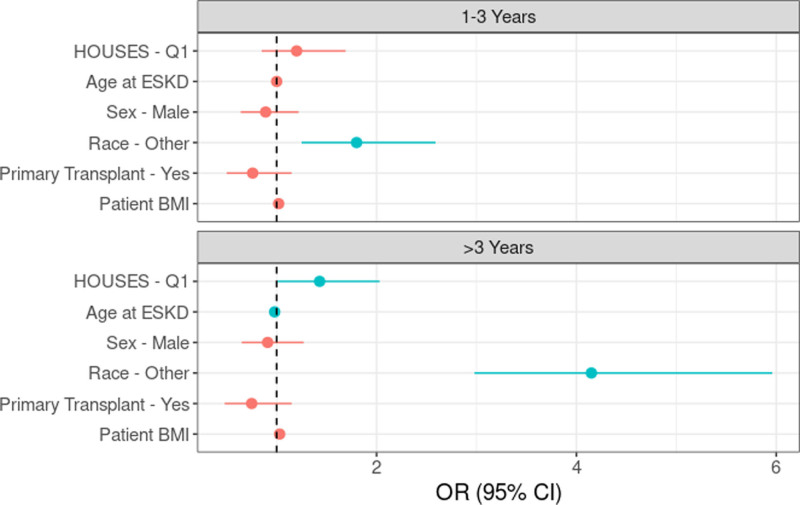
Pretransplant dialysis duration (reference <1 y duration). BMI, body mass index; CI, confidence interval; ESKD, end-stage kidney disease; HOUSES, HOUsing-based SocioEconomic Status measure; OR, odds ratio.

After adjusting for age at kidney failure, sex, race, prior transplantation, and recipient BMI, Q1 recipients were more likely to have been on pretransplant dialysis for >3 y compared with Q2–Q4 recipients (aOR: 0.43; 95% CI, 1.01-2.03; *P* = 0.046; Table 4; Figure 2). When the analysis was performed using the HOUSES quartiles, Q1 recipients (aOR: 1.87; 95% CI, 1.16-3.02; *P* = 0.011) had significantly higher odds of being on dialysis for >3 y compared with Q4 recipients. However, the difference between Q2 versus Q4 (aOR: 1.60; 95% CI, 0.97-2.63; *P* = 0.06) and Q3 versus Q4 recipients (aOR: 1.29; 95% CI, 0.78-2.15; *P* = 0.32) did not achieve statistical significance (**Table S2, SDC,** http://links.lww.com/TXD/A718).

**TABLE 4.** Pretransplant dialysis duration for Q1 vs Q2–Q4 recipients (reference <1 y)

Variables	1–3 y on dialysis			>3 y on dialysis		
	OR	95% CI	*P*	OR	95% CI	*P*
HOUSES						
Q1	1.20	0.85-1.69	0.294	1.43	1.01-2.03	0.046
Q2–Q4	Reference	–	–	Reference	–	
Age at kidney loss, y	1.00	0.99-1.01	0.752	0.98	0.96-0.99	<0.001
Sex						
Female	Reference	–	–	Reference	–	–
Male	0.89	0.64-1.22	0.464	0.91	0.65-1.27	0.57
Race						
Non-Hispanic White	Reference	–	–	Reference	–	–
Other	1.80	1.25-2.59	0.002	4.15	2.98-5.96	<0.001
Primary transplant						
No	Reference	–	–	Reference	–	–
Yes	0.76	0.50-1.15	0.197	0.75	0.48-1.15	0.19
Patient BMI kg/m^2^	1.02	0.99-1.06	0.155	1.03	1.00-1.07	0.05

BMI, body mass index; CI, confidence interval; HOUSES, HOUsing-based SocioEconomic Status measure; OR, odds ratio.

### Death-censored Graft Loss

**FIGURE 3.**
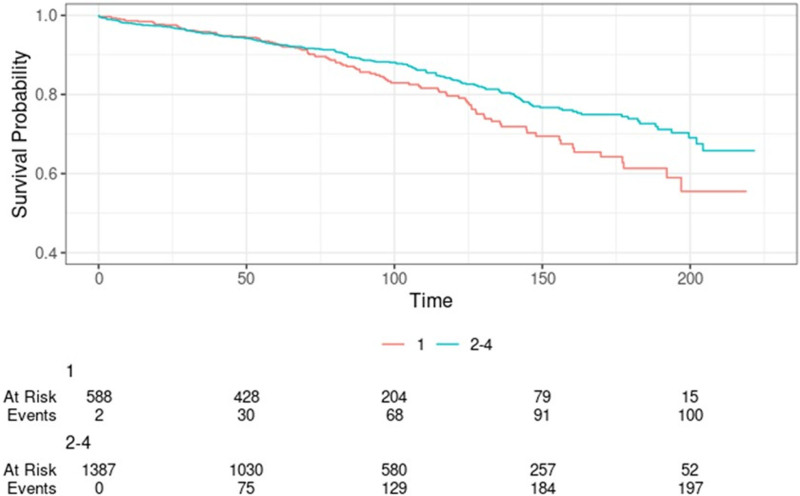
Death-censored graft survival by HOUSES (Q1 vs Q2–Q4; log-rank *P* = 0.02). HOUSES, HOUsing-based SocioEconomic Status measure.

We observed a significant difference in 10-y graft survival between Q1 and Q2–Q4 adult kidney transplant recipients (10-y graft survival: 79.6% versus 83.6%; log-rank *P* = 0.02; Figure 3). We also observed a trend toward higher 10-y graft survival with increasing HOUSES quartile (Q1 [79.6%], Q2 [81.7%], Q3 [83.0%], and Q4 [86.9%]; *P* = 0.06).

On unadjusted analysis by quartiles, Q1 recipients were at a significantly higher risk of death-censored graft loss compared with Q4 recipients (HR: 1.45; 95% CI, 1.04-2.02; *P* = 0.03). However, the difference between Q2 versus Q4 (HR: 1.23; 95% CI, 0.87-1.73; *P* = 0.25) and Q3 versus Q4 (HR: 1.00; 95% CI, 0.70-1.44; *P* = 0.99) did not achieve statistical significance.

After adjusting for age at transplant, sex, race, pretransplant dialysis, donor type, HLA mismatch, and prior transplantation, Q1 recipients were at a significantly higher risk of death-censored graft loss compared with Q2–Q4 recipients (adjusted HR: 1.36; 95% CI, 1.02-1.82; *P* = 0.036; Table 5). However, when the analysis was performed using HOUSES quartiles, the statistical significance was lost (**Table S3, SDC,** http://links.lww.com/TXD/A718).

**TABLE 5.** Death-censored graft loss– multivariable analysis

Variables	HR	95% CI	*P*
HOUSES			
Q1	1.37	1.02-1.82	0.036
Q2–Q4		Reference	
Age at transplant, y			
18–35		Reference	–
35–<50	0.69	0.46-1.05	0.081
50–<65	0.76	0.52-1.13	0.17
+65	0.95	0.58-1.50	0.82
Sex			
Female		Reference	–
Male	0.99	0.75-1.30	0.93
Race			
Non-Hispanic White		Reference	–
Other	1.60	1.20-2.13	0.002
Pretransplant dialysis			
No		Reference	–
Yes	2.17	1.48-3.18	<0.001
Donor source			
Deceased		Reference	–
Living	0.63	0.46-0.87	0.005
HLA mismatch	1.08	0.99-1.17	0.053
Prior transplant			
Yes		Reference	–
No (primary transplant)	0.66	0.47-0.93	0.016

CI, confidence interval; HOUSES, HOUsing-based SocioEconomic Status measure; HR, hazard ratio.

### Death-censored Graft Loss Stratified by Insurance Type

**FIGURE 4.**
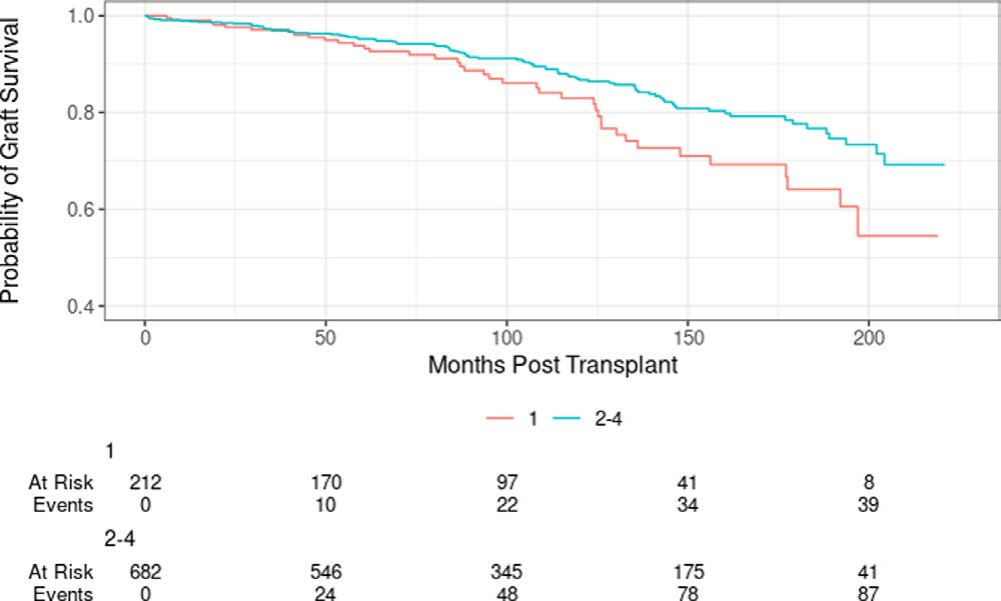
Private insurance patients death-censored graft survival by HOUSES (Q1 vs Q2–Q4; log-rank *P* = 0.022). HOUSES, HOUsing-based SocioEconomic Status measure.

**FIGURE 5.**
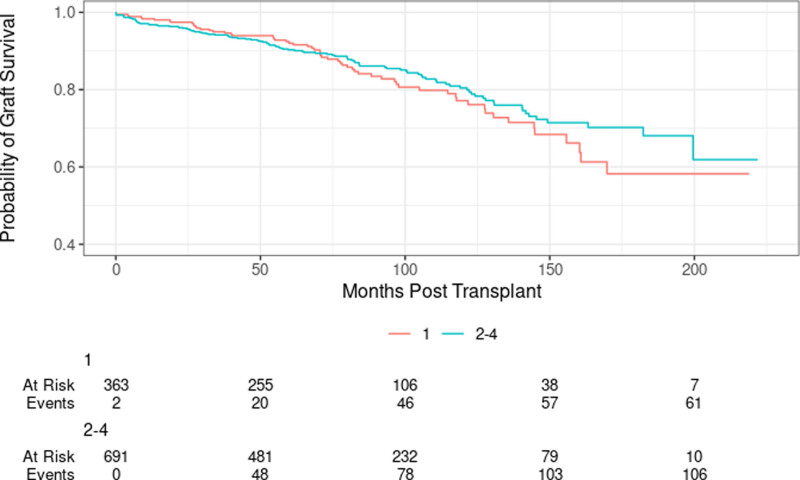
Public insurance patients death-censored graft survival by HOUSES (Q1 vs Q2–Q4; log-rank *P* = 0.433). HOUSES, HOUsing-based SocioEconomic Status measure.

Q1 recipients had significantly lower graft survival compared with Q2–Q4 recipients in the privately insured group (Figure 4). However, we found no difference among those on public insurance (Figure 5).

## DISCUSSION

This study examines the association between a new individual-level SES measure (HOUSES) and kidney transplant access and outcomes among a cohort of transplant recipients at a single center. We found low SES, as measured by HOUSES, to be significantly associated with a reduced likelihood of preemptive transplantation, an increased risk of graft loss, and a greater likelihood of pretransplant dialysis for >3 y compared with those with higher SES. The results corroborate the findings of our earlier study, demonstrating a strong association between HOUSES and graft survival. These studies offer crucial insights into the impact of SDOH on access to kidney transplants and transplant outcomes. By identifying a high-risk population, HOUSES has the potential to inform effective clinical and social care interventions to improve access to and outcomes of kidney transplants.

Our present study validates the findings of our earlier study, illustrating an association between HOUSES and graft survival.^25^ In the literature, the results on the association between SES and graft survival have been inconsistent. Although some studies found private insurance, higher level of education, and skilled occupation to be associated with improved graft survival,^19,33,34^ other studies found no association, likely due to the use of area-level measures of SES, such as neighborhood poverty levels.^35-37^ As shown in our prior study, these inconsistent associations are likely to be due to imprecision or misclassification bias of the conventional SES measures.^25^ For example, in our previous study, HOUSES was associated with posttransplant graft loss, but area-level SES measure and educational attainment were not.^25^ Educational attainment often used in kidney transplant literature may reflect patients’ health literacy and current or future job potential but not necessarily current SES, especially for older patients, resulting in failure to unveil health disparities partly due to its static nature.^25^ In the recent literature, household wealth is becoming an important SES measure instead of static educational level or income. Pollack et al argued that in most studies, greater wealth is associated with better health, even after adjusting for other SES measures.^38^ In this respect, HOUSES reflects household wealth and may better capture the underlying constructs of SES, specifically the capacity to access desired resources, including human, materialistic, and social resources, which can be critical determinants of transplant outcomes.

Our study indicated that patients with lower SES, as measured by HOUSES, were at increased risk of graft loss independent of age at transplant, sex, race, pretransplant dialysis, donor type, HLA mismatch, and prior transplantation. HOUSES predicted graft survival among recipients who are typically approved for the transplant after a comprehensive clinical and SDOH evaluation. A greater proportion of deceased-donor transplants and longer pretransplant dialysis exposure among patients in the Q1 stratum might have played a role in mediating the association between HOUSES and graft loss, but these are unlikely to account for it fully as we adjusted for these factors in the multivariable model. It is possible that HOUSES identified the group of patients who struggled to continue posttransplant immunosuppressive medication after losing Medicare coverage after the first 3 y.^39^ The limited coverage for the initial 3 y might also have dissuaded low SES patients from seeking a timely transplant, fearing the inability to afford the medications beyond coverage, contributing to disparities in transplant access. Fortunately, as of January 2023, transplant recipients are eligible for lifetime Medicare coverage of posttransplant immunosuppressive medications.^39^ However, our study predates the favorable change in Medicare policy.

One of the goals of the Healthy People 2020 Program is to improve preemptive transplant access.^40^ A recently published retrospective study by King et al^28^ of 157 073 deceased-donor kidney transplants performed in the United States between 2000 and 2018 documented a preemptive transplant rate of 9.3%. The higher preemptive transplant rates in our study cohort (30%) compared with the study by King et al could be partly due to the less diverse patient population with a higher proportion of White patients and partly due to transplantation at a single center with a longstanding transplant program with an emphasis on living-donor transplantation. Although the overall rate was relatively high, we found significant socioeconomic disparities in access to preemptive transplantation. Congruent with our findings, studies have identified sociodemographic barriers to preemptive transplantation, including race, insurance type, and education level.^13,28,41^ Considering the relationship between race and SES,^42^ one may question if the association between SES and preemptive transplants is confounded by race. However, we found the effect of SES on preemptive transplantation to be independent of race. SES may mediate its effect on preemptive transplants through several factors, including differential access to nephrology care by SES,^43^ delayed,^44^ or no referral^45^ of patients with low SES for transplant evaluation, and inadequate education regarding the benefits of transplantation for those with lower SES. The latter is because SES is intricately linked to health literacy.^15^ Because early referrals for transplant evaluation are associated with higher rates of preemptive transplants,^46^ we suggest that transplant candidates with limited ability to access resources, as indicated by a lower HOUSES index, be considered for transplant referral at relatively higher residual kidney function (for instance, at a glomerular filtration rate of >20 mL/min/1.73 m^2^) and through a designated care coordination program. Early evaluation and care coordination for this vulnerable population will allow timely implementation of interventions to address medical and psychosocial barriers to transplant.

Preemptive deceased-donor kidney transplants require that transplant candidates be preemptively listed on the deceased-donor waitlist (listing before initiation of dialysis). According to the 2022 Scientific Registry of Transplant Recipients annual report, only 22.7% of the candidates listed in 2021 were listed preemptively. Additionally, 16.7% of the waitlisted patients had been on dialysis for >6 y before listing.^47^ Using Scientific Registry of Transplant Recipients data, Keith et al^48^ found Medicare insurance, non-White race, and low educational attainment to be associated with delayed preemptive listing and prolonged exposure to prelisting dialysis. Aligned with these findings, we found socioeconomic disparities in pretransplant dialysis duration. Patients with lower HOUSES index were more likely to be on dialysis for >3 y. Because pretransplant dialysis duration is a modifiable risk factor associated with graft survival,^5^ identification of patients at risk of prolonged dialysis can inform strategic planning to decrease pretransplant dialysis exposure. In this respect, HOUSES can be a validated, standardized, objective, patient contact-free, and nationally available individual SES measure to identify patients who could benefit from relaxed listing criteria (for instance, higher estimated glomerular filtration rate at listing). The conventional measures of SES may not effectively capture or identify this vulnerable patient population, as demonstrated in our previous study.^25^

This study had a few limitations. Given the retrospective nature, we could not account for unknown confounders. Due to sample limitations (a higher proportion of non-Hispanic White patients), we could not examine an interaction between HOUSES and race. Due to a small number of death events, the effect of HOUSES on patient survival could not be examined. A more extended follow-up study is needed to study the association between HOUSES and mortality effectively. Due to the small sample size, we could not demonstrate a clear association between HOUSES quartiles and graft survival. HOUSES was computed on the basis of addresses at the time of transplant. Hence, our access to transplant analysis was based on the assumption that SES was the same at the time of referral as it was at the time of transplant. This study corroborates the findings of our earlier study, indicating a link between HOUSES and graft survival while addressing the limitations of our previous study by using a significantly larger and more diverse transplant cohort in a metropolitan city.^25^ As HOUSES is available nationwide, a multicenter national study should be conducted to study the effects of SES on transplant access and outcomes, overcoming the limitations of single-center studies.

In conclusion, this study highlights significant socioeconomic disparities among transplant recipients in kidney transplant access, pretransplant dialysis duration, and graft survival. The study also demonstrates the utility of HOUSES in identifying socioeconomic inequalities in access to and outcomes of a kidney transplant. The findings emphasize the need for targeted interventions aimed at addressing SDOH needs and alleviating these disparities. Early referral, preemptive listing strategies, and social interventions guided by HOUSES may be effective strategies to mitigate disparities. Future research should focus on evaluating the efficacy of HOUSES-based interventions in improving access to and outcomes of kidney transplantation.

## Supplementary Material


