# Supplementary material for: Socioeconomic Inequities in Preemptive Kidney Transplantation and Graft Survival: An Innovative Approach to Identifying Disparities in Kidney Transplantation
Source: Transplant Direct. 2024 Dec 18;11(1):e1734. doi: 10.1097/TXD.0000000000001734 (PMC11658720; doi:10.1097/TXD.0000000000001734)
Supplement: Supplementary file 1 [file txd-11-e1734-s001.pdf]

Appendix

Table 1. Publications showing health disparities by HOUSES (adults) (39 outcomes)		
**Study**	**Outcomes**	**Adjusted effect size for Q1 (lowest SES by HOUSES index) (95% CI)[a]**
**1. Chronic conditions**		
Ahn, 2023[1]	**Alcohol-associated hepatitis**	Distribution of HOUSES: 39.9% (Q1), 12.5%% (Q2), 12.3% (Q3), and 17.1% (Q4)
Tadese, 2022[2]	**Severe obesity**	OR = 1.33 (0.99-1,80)
Greenwood, 2022[3]	**Diabetic control**	OR = 0.83 (0.70-0.98)
Rusk, 2022[4]	**Indigenous Smoking Behaviors**	Distribution of HOUSES in indigenous cohort (vs. match cohort): 39.9% (vs. 26.7%) for Q1, 12.5%% (vs. 24.3%) for Q2, 10.4% (vs. 20.6%) for Q3, and 8.0% (vs. 24.8%) for Q4
Rusk, 2022[5]	**Smoking Behaviors Among Indigenous Pregnant People**	Distribution of HOUSES in indigenous cohort (vs. match cohort): 40.4% (vs. 35.2%) for Q1, 33.3% (vs. 28.1%) for Q2, 12.3% (vs. 23.0%) for Q3, and 14.0% (vs. 13.8%) for Q4
Angstman, 2021[6]	**Persistent depressive symptoms**	OR = 1.31 (1.06-1.61)
	**Remission of depressive symptoms**	OR = 0.78 (0.65-0.95)
Thacher, 2020[7]	**25-Hydroxyvitamin D Level**	Estimate = 0.28 (0.21-0.35)
Stevens, 2020[8]	**Kidney transplantation failure**	HR = 2.0 (1.04-3.84)
Ryan, 2020[9]	**Post-Glioma mortality**	HR = 1.61 (1.05-2.5)
Patten, 2020[10]	**Mood disorder and obesity**	Distribution of HOUSES in rural (vs. urban): 11% (vs. 28%) for Q1, 21% (vs. 27%) for Q2, 28% (vs. 25%) for Q3, 40% (20%) for Q4
Takahashi, 2016[11]	**All-cause hospitalization**	HR = 1.53 (1.18-1.98)
	**Multiple chronic conditions prevalence**	OR = 2.4 (2.0-3.1)
Wi, 2016[12]	**Coronary heart disease prevalence**	OR = 1.35 (1.25-1.44)
	**Asthma prevalence**	OR = 1.28 (1.20-1.36)
	**Diabetes prevalence**	OR = 1.78 (1.66-1.92)
	**Hypertension prevalence**	OR = 1.56 (1.47-1.63)
	**Mood disorder prevalence**	OR = 1.63 (1.58-1.72)
Ghawi, 2015[13]	**Rheumatoid Arthritis incidence**	OR = 1.06 (1.02-1.09)
	**Post-Rheumatoid Arthritis mortality**	HR = 1.58 (1.05-2.36)
Bang, 2014[14]	**Post–Myocardial Infarction mortality**	HR = 1.86 (1.07-3.24)
**2. Acute conditions**		
Wi, 2023[15]	**Subject recruitment**	Distribution of HOUSES in Standard mailing (TESRS): 16.1% (vs. 12.9% for Q1, 9.7 (vs. 12.9%) for Q2, 45.2% (vs. 45.2%) for Q3, and 29.0% (vs. 29.0%) for Q4
Zurek, 2022[16]	**Hospital readmission**	OR* = 0.90 (0.83-0.98) for Q3
Vachon, 2022[17]	**COVID-19 asymptomatic rates**	HR* = 1.22 (0.86-1.7) for Q4
Juhn 2021[18]	**COVID-19 incidence in urban**	Identified geographical hotspots taking into account HOUSES

Wheeler 2021[19]	**COVID-19 incidence in rural**	Identified geographical hotspots taking into account HOUSES
Aul, 2020[20]	**Osteoporotic fracture incidence**	HR = 1.05 (1.04-1.08)
Barwise, 2020[21]	**Mortality rates (>50 years of age)**	HR = 1.38 (1.07-1.78)
Ryu, 2017[22]	**Accidental falls incidence**	HR = 1.72 (1.31-2.27)
**3. Behavioral risk factors and others**		
Felzer, 2023[23]	**Influenza vaccination in solid organ transplant patients**	RR* = 1.14 (1.008-1.30) for Q3
Juhn, 2021[24]	**Adherence to Public Health Measures Mitigates the Risk of COVID-19 Infection**	Used as a covariate for basic characteristics 12.3% (Q1), 25.5% (Q2), 28.3% (Q3), and 33.9% (Q4)
MacLaughlin, 2020[25]	**HPV Vaccination (initiation and completion)**	RR = 1.15 [1.03–1.28] for Q4 (initiation)<br>RR = 1.32 [1.21–1.44] for Q4 (completion)
Barwise, 2019[26]	**Advance Care Planning and Nursing Home Utilization**	OR* = 0.77 (0.63-0.93) for advance directives<br>OR* = 0.60 (0.50-0.72) for discharge to home
Ryu, 2018[27]	**Inconsistency of self-reported disease prevalence in survey results**	OR = 1.46 (1.17-1.84)
Barwise, 2018[28]	**Rates of social work consultation in end-of-life care**	OR = 1.46 (1.18-1.79)
	**Advance directives**	OR =1.29 (1.07-1.58)
Wi, 2016[29]	**Smoking status**	OR = 2.56 (1.14-5.55)

[a] Highest HOUSES group as a reference, except Zurek et al 2022* and Felzer et al 2022* which used lowest HOUSES group, Vachon et al 2022* which used 2nd quartile of HOUSES group, and Barwise et al 2019* which used 2nd-4th quartile of HOUSES group as a reference

Table 2. Publications showing health disparities by HOUSES (children) (23 outcomes)		
**Study**	**Outcomes**	**Adjusted effect size for Q1 (lowest SES by HOUSES index) (95% CI)[a]**
**1. Chronic conditions**		
Skolnick 2023[30]	**Severe Obesity**	OR = 2.78 (1.91-4.06)
Rodriguez, 2023[31]	**Acne prevalence**	Distribution of HOUSES in acne cases (vs. matched controls): 42.2% (vs. 32.7%) for Q1, 22.8% (vs. 25.5%) for Q2, 17.4% (vs. 20.1%) for Q3, and 17.5% (21.7%) for Q4
Bjur 2019[32]	**Asthma prevalence**	OR = 1.14 (1.03-1.29)
	**Epilepsy prevalence**	OR = 1.88 (1.21-2.85)
	**Mood disorders prevalence**	OR =1.38 (1.19-1.61)
Bjur 2019[33]	**Multiple complex chronic conditions prevalence**	5-year prevalence in 2004:<br>Q1 (1,443), Q2 (1,435), Q3 (1,134), Q4 (1,124)
Ryu 2016[34]	**Overweight prevalence**	OR = 2.08 (1.06-4) (OC) [b]<br>OR = 2.22 (1.12-4.34) (JC) [b]
	**Low birth weight prevalence**	OR = 1.28 (0.49-3.33) (OC) [b]<br>OR = 1.85 (0.82-2.94) (JC) [b]
	**Household smoking status**	OR = 4.16 (2.04-9.09) (OC) [b]<br>OR = 3.84 (2.27-6.25) (JC) [b]
Lynch 2015[35]	**Overweight/Obesity (HOUSES as covariate)**	Mean (SD) (p<0.0001)<br>Healthy weight: 1.7 (3.9)<br>Overweight: 1.0 (3.8)<br>Obesity: 0.1 (3.4)
Harris 2014[36]	**Poorly controlled asthma status**	OR = 4.76 (1.12-20)
Butterfield 2011[37]	**Adverse self-rated health**	OR = 4.76 (1.96-12.5) (OC) [b]<br>OR = 1.01 (0.24-4.0) (JC) [b]
Juhn 2011[38]	**Overweight prevalence**	OR=2.56 (p=0.008) (OC) [b]<br>OR=2.04 (p=0.07) (JC) [b]
	**Low birth weight prevalence**	OR=1.58 (p=0.330) (OC) [b]<br>OR=2.38 (p=0.018) (JC) [b]
	**Household smoking status prevalence**	OR=2.56 (p=0.007) (OC) [b]<br>OR=4.16 (p<.001) (JC) [b]
**2. Acute conditions**		
Patel 2020[22]	**Adverse childhood events risk (HOUSES as covariate)**	Proportion of outcomes by HOUSES quartile<br>Q1 (4.6%), Q2 (3.5%), Q3 (3.0%), Q4 (2.1%)
Bjur 2019[32]	**Bronchiolitis prevalence**	OR = 1.44 (1.28-1.63)
	**Pneumonia prevalence**	OR = 1.13 (1.01-1.28)
	**Urinary Tract Infection prevalence**	OR = 1.51 (1.28-1.78)
	**Adverse childhood experiences prevalence**	OR = 1.85 (1.51-2.27)
Johnson 2013[39]	**Invasive pneumococcal disease risk**	OR = 4.54 (1.12-20)
**3. Behavioral risk factors**		
Maclaughlin 2020[25]	**HPV vaccine initiation**	Rates: 0.86 (0.78-0.97)
	**HPV vaccine completion**	Rates: 0.75 (0.69-0.82)

Hammer 2016[40]	**Pertussis vaccine up-to-date status**	OR = 0.27 (0.10-0.74)
**4. AI bias**		
Juhn 2022[41]	**Performance of predicting risk of asthma exacerbation**	Balance error rate [(FPR+FNR)/2]<br>Q1 = 0.53, Q2-4: 0.39 (Ratio: 1.35)
[a] Highest HOUSES group as a reference; [b] OC: Olmsted county, Minnesota JC: Jackson county, Missouri		

1. Ahn JC, Wi CI, Buryska S, et al. Disproportionate increases in alcohol-associated hepatitis incidence in women and individuals of low socioeconomic status: A population-based study using the Rochester epidemiology project database. Hepatology Communications 2023;7.
2. Tadese K, Ernst V, Weaver AL, et al. Association of Perinatal Factors With Severe Obesity and Dyslipidemia in Adulthood. J Prim Care Community Health 2022;13:21501327211058982.
3. Greenwood J, Zurek KI, Grimm JM, et al. Association of a housing based individual socioeconomic status measure with diabetic control in primary care practices. Prim Care Diabetes 2022;16:78-83.
4. Rusk AM, Giblon RE, Chamberlain AM, et al. Indigenous Smoking Behaviors in Olmsted County, Minnesota: A Longitudinal Population-Based Study. Mayo Clinic Proceedings 2022;97:1836-1848.
5. Rusk AM, Giblon RE, Chamberlain AM, et al. Smoking Behaviors Among Indigenous Pregnant People Compared to a Matched Regional Cohort. Nicotine & Tobacco Research 2023;25:889-897.
6. Angstman KB, Wi CI, Williams MD, et al. Impact of socioeconomic status on depression clinical outcomes at six months in a Midwestern, United States community. Journal of Affective Disorders 2021;292:751-756.
7. Thacher TD, Dudenkov DV, Mara KC, et al. The relationship of 25-hydroxyvitamin D concentrations and individual-level socioeconomic status. J Steroid Biochem Mol Biol 2020;197:105545.
8. Stevens MA, Beebe TJ, Wi C-I, et al. HOUSES index as an innovative socioeconomic measure predicts graft failure among kidney transplant recipients. Transplantation 2020;Online First.
9. Ryan CS, Juhn YJ, Kaur H, et al. Long-term incidence of glioma in Olmsted County, Minnesota, and disparities in postglioma survival rate: a population-based study. Neurooncol Pract 2020;7:288-298.
10. Patten CA, Juhn YJ, Ryu E, et al. Rural-urban health disparities for mood disorders and obesity in a midwestern community. Journal of Clinical and Translational Science 2020;4:408-415.
11. Takahashi PY, Ryu E, Hathcock MA, et al. A novel housing-based socioeconomic measure predicts hospitalisation and multiple chronic conditions in a community population. J Epidemiol Community Health 2016;70:286-91.
12. Wi CI, St Sauver JL, Jacobson DJ, et al. Ethnicity, Socioeconomic Status, and Health Disparities in a Mixed Rural-Urban US Community-Olmsted County, Minnesota. Mayo Clin Proc 2016;91:612-22.
13. Ghawi H, Crowson CS, Rand-Weaver J, et al. A novel measure of socioeconomic status using individual housing data to assess the association of SES with rheumatoid arthritis and its mortality: a population-based case-control study. BMJ Open 2015;5:e006469.
14. Bang DW, Manemann SM, Gerber Y, et al. A novel socioeconomic measure using individual housing data in cardiovascular outcome research. Int J Environ Res Public Health 2014;11:11597-615.
15. Wi CI, King KS, Ryu E, et al. Application of Innovative Subject Recruitment System for Batch Enrollment: A Pilot Study. Journal of Primary Care and Community Health 2023;14.
16. Zurek KI, Boswell CL, N EM, et al. Association of Early and Late Hospital Readmissions with a Novel Housing-Based Socioeconomic Measure. Health Serv Res Manag Epidemiol 2022;9:23333928221104644.
17. Vachon CM, Norman AD, Prasad K, et al. Rates of Asymptomatic COVID-19 Infection and Associated Factors in Olmsted County, Minnesota, in the Prevaccination Era. Mayo Clin Proc Innov Qual Outcomes 2022;6:605-617.

18. Juhn YJ, Wheeler P, Wi CI, et al. Role of Geographic Risk Factors in COVID-19 Epidemiology: Longitudinal Geospatial Analysis. Mayo Clin Proc Innov Qual Outcomes 2021;5:916-927.
19. Wheeler PH, Patten CA, Wi CI, et al. Role of geographic risk factors and social determinants of health in COVID-19 epidemiology: Longitudinal geospatial analysis in a midwest rural region. Journal of Clinical and Translational Science 2021;6.
20. Aul AJ, Dudenkov DV, Mara KC, et al. The relationship of 25-hydroxyvitamin D values and risk of fracture: a population-based retrospective cohort study. Osteoporos Int 2020;31:1787-1799.
21. Barwise A, Wi CI, Frank R, et al. An Innovative Individual-Level Socioeconomic Measure Predicts Critical Care Outcomes in Older Adults: A Population-Based Study. J Intensive Care Med 2020:885066620931020.
22. Ryu E, Juhn YJ, Wheeler PH, et al. Individual housing-based socioeconomic status predicts risk of accidental falls among adults. Ann Epidemiol 2017;27:415-420.e2.
23. Felzer JR, Rutten LFJ, Wi CI, et al. Disparities in vaccination rates in solid organ transplant patients. Transplant Infectious Disease 2023;25.
24. Juhn YJ, Wi CI, Ryu E, et al. Adherence to Public Health Measures Mitigates the Risk of COVID-19 Infection in Older Adults: A Community-Based Study. Mayo Clin Proc 2021;96:912-920.
25. MacLaughlin KL, Jacobson RM, Sauver JLS, et al. An innovative housing-related measure for individual socioeconomic status and human papillomavirus vaccination coverage: A population-based cross-sectional study. Vaccine 2020;38:6112-6119.
26. Barwise A, Juhn YJ, Wi CI, et al. An Individual Housing-Based Socioeconomic Status Measure Predicts Advance Care Planning and Nursing Home Utilization. American Journal of Hospice & Palliative Medicine 2019;36:362-369.
27. Ryu E, Olson JE, Juhn YJ, et al. Association between an individual housing-based socioeconomic index and inconsistent self-reporting of health conditions: a prospective cohort study in the Mayo Clinic Biobank. BMJ Open 2018;8:e020054.
28. Barwise A, Juhn YJ, Wi CI, et al. An Individual Housing-Based Socioeconomic Status Measure Predicts Advance Care Planning and Nursing Home Utilization. Am J Hosp Palliat Care 2018.
29. Wi CI, Gauger J, Bachman M, et al. Role of individual-housing-based socioeconomic status measure in relation to smoking status among late adolescents with asthma. Ann Epidemiol 2016;26:455-60.
30. Skolnick V, Rajjo T, Thacher T, et al. Association of Weight Trajectory with Severe Obesity: A Case-Control Study. Childhood Obesity 2023.
31. Baisi KRE, Weaver AL, Wi CI, et al. Socioeconomic status, race, and preadolescent acne: A population-based retrospective cohort analysis in a mixed rural-urban community of the United States (Olmsted County, Minnesota). Pediatric Dermatology 2023;40:460-465.
32. Bjur KA, Wi CI, Ryu E, et al. Socioeconomic Status, Race/Ethnicity, and Health Disparities in Children and Adolescents in a Mixed Rural-Urban Community-Olmsted County, Minnesota. Mayo Clin Proc 2019;94:44-53.
33. Bjur KA, Wi CI, Ryu E, et al. Epidemiology of Children With Multiple Complex Chronic Conditions in a Mixed Urban-Rural US Community. Hosp Pediatr 2019;9:281-290.
34. Ryu E, Wi CI, Crow SS, et al. Assessing health disparities in children using a modified housing-related socioeconomic status measure: a cross-sectional study. BMJ Open 2016;6:e011564.
35. Lynch BA, Finney Rutten LJ, Jacobson RM, et al. Health Care Utilization by Body Mass Index in a Pediatric Population. Acad Pediatr 2015;15:644-50.
36. Harris MN, Lundien MC, Finnie DM, et al. Application of a novel socioeconomic measure using individual housing data in asthma research: an exploratory study. NPJ primary care respiratory medicine 2014;24:14018.

37. Butterfield MC, Williams AR, Beebe T, et al. A two-county comparison of the HOUSES index on predicting self-rated health. J Epidemiol Community Health 2011;65.
38. Juhn YJ, Beebe TJ, Finnie DM, et al. Development and initial testing of a new socioeconomic status measure based on housing data. J Urban Health 2011;88.
39. Johnson MD, Urm SH, Jung JA, et al. Housing data-based socioeconomic index and risk of invasive pneumococcal disease: an exploratory study. Epidemiology and infection 2013;141:880-7.
40. Hammer R, Capili C, Wi C-I, et al. A new socioeconomic status measure for vaccine research in children using individual housing data: a population-based case-control study. BMC Public Health 2016;16:1-9.
41. Juhn YJ, Ryu E, Wi CI, et al. Assessing socioeconomic bias in machine learning algorithms in health care: a case study of the HOUSES index. Journal of the American Medical Informatics Association 2022;29:1142-1151.

Table S1: Preemptive Kidney Transplantation - Adjusted Analysis with HOUSES QUARTILES

Variables	OR	(95% Confidence Interval)	P value
**HOUSES**			
Q1	0.53	(0.38, 0.74)	<0.001
Q2	0.57	(0.40, 0.81)	0.002
Q3	0.66	(0.47, 0.93)	0.017
Q4		Ref	
**Age at Transplant**	1.00	(0.99, 1.01)	0.488
**Sex**			
Female		Ref	
Male	0.72	(0.56, 0.92)	0.009
**Race**			
Non-Hispanic white		Ref	
Other	0.36	(0.27, 0.49)	<0.001
**Primary Transplant**			
No		Ref	
Yes	1.28	(0.92, 1.77)	0.140
**Patient BMI (kg/m^2)**	1.01	(0.99, 1.04)	0.343

OR: odds ratio

*Note: This is a mixed effects logistic regression model. The random effect is county to account for the HOUSES index being standardized at the county level. The binary outcome is preemptive transplant or not.

Table S2: Pretransplant dialysis duration (ref <1 year) with HOUSES QUARTILES.

	1 – 3 Years on Dialysis			>3 Years on Dialysis		
**Variables**	**OR**	**(95% Confidence Interval)**	**P value**	**OR**	**(95% Confidence Interval)**	**P value**
**HOUSES**						
Q1	1.45	(0.92, 2.27)	0.106	1.87	(1.16, 3.02)	0.011
Q2	1.31	(0.82, 2.08)	0.254	1.60	(0.97, 2.63)	0.063
Q3	1.29	(0.81, 2.05)	0.278	1.29	(0.78, 2.15)	0.319
Q4	Ref	--		Ref	--	
**Age at kidney loss,yr**	1.00	(0.99, 1.01)	0.820	0.98	(0.97, 0.99)	<0.001
**Sex**						
Female	Ref	--	--	Ref	--	--
Male	0.90	(0.65, 1.25)	0.524	0.92	(0.65, 1.29)	0.611
**Race**						
Non-Hispanic white	Ref	--	--	Ref	--	--
Other	1.80	(1.25, 2.59)	0.002	4.14	(2.88, 5.94)	<0.001
**Primary transplant**						
No	Ref	--	--	Ref	--	--
Yes	0.76	(0.50, 1.6)	0.200	0.76	(0.49, 1.17)	0.208
**Patient BMI kg/m^2**	1.02	(0.99, 1.06)	0.159	1.03	(1.00, 1.07)	0.058

OR: odds ratio

Table S3: Death-censored Graft Loss– Multivariable Analysis with HOUSES QUARTILES

**Variables**	**HR**	**(95% Confidence Interval)**	**P value**
**HOUSES**			
Q1	1.33	(0.88, 2.04)	0.176
Q2	1.02	(0.65, 1.58)	0.948
Q3	0.93	(0.59, 1.48)	0.771
Q4		Ref	
**Age at transplant, yr**			
18-35		Ref	-
35-<50	0.69	(0.46, 1.05)	0.080
50-<65	0.77	(0.52, 1.13)	0.182
+65	0.94	(0.60, 1.50)	0.808
**Sex**			
Female		Ref	-
Male	0.99	(0.74, 1.31)	0.916
**Race**			
Non-Hispanic White		Ref	-
Race Other	1.59	(1.19, 2.13)	0.002
**Pretransplant dialysis**			
No		Ref	-
Yes	2.17	(1.47, 3.18)	<0.001
**Donor Source**			
Deceased		Ref	-
Living	0.64	(0.47, 0.93)	0.005
**HLA Mismatch**	1.08	(1.00, 1.17)	0.055
**Prior transplant**			
Yes		Ref	-
No (primary transplant)	0.66	(0.47, 0.93)	0.017

HR: Hazard ratio
